# Photoconductivity of acid exfoliated and flash-light-processed MoS_2_ films

**DOI:** 10.1038/s41598-018-21688-0

**Published:** 2018-02-19

**Authors:** Renyun Zhang, Magnus Hummelgård, Viviane Forsberg, Henrik Andersson, Magnus Engholm, Thomas Öhlund, Martin Olsen, Jonas Örtegren, Håkan Olin

**Affiliations:** 1 Department of Natural Sciences, Mid Sweden University, Holmgatan 10, SE, 85170 Sundsvall, Sweden; 2 Department of Electronic Design, Mid Sweden University, Holmgatan 10, SE, 85170 Sundsvall, Sweden

## Abstract

MoS_2_ has been studied intensively during recent years as a semiconducting material in several fields, including optoelectronics, for applications such as solar cells and phototransistors. The photoresponse mechanisms of MoS_2_ have been discussed but are not fully understood, especially the phenomenon in which the photocurrent slowly increases. Here, we report on a study of the photoresponse flash-light-processed MoS_2_ films of different thicknesses and areas. The photoresponse of such films under different light intensities and bias voltages was measured, showing significant current changes with a quick response followed by a slow one upon exposure to pulsed light. Our in-depth study suggested that the slow response was due to the photothermal effect that heats the MoS_2_; this hypothesis was supported by the resistivity change at different temperatures. The results obtained from MoS_2_ films with various thicknesses indicated that the minority-carrier diffusion length was 1.36 µm. This study explained the mechanism of the slow response of the MoS_2_ film and determined the effective thickness of MoS_2_ for a photoresponse to occur. The method used here for fabricating MoS_2_ films could be used for fabricating optoelectronic devices due to its simplicity.

## Introduction

Molybdenum disulfide (MoS_2_) is a layered transition metal dichalcogenide (TMDC), in which the molecular layers are stacked via van der Waals forces^1^. This 2D semiconductor material has attracted increasing interest due to its promising applications^2^, such as photovoltaic and photocatalytic applications^3^.

Photoconductivity has been measured on both single-layer and multilayer MoS_2_. Lu and co-workers studied the photoelectrical properties of single- and four-layer MoS_2_, indicating a higher photosensitivity of the laser-modified single layer than of the pristine multilayer MoS_2_^4^. Kis’s group^5^ has reported an 880 AW^−1^ photoresponsivity of a single-layer MoS_2_ on a silicon gated device. Both of their devices were built on a single MoS_2_ flake, making the process difficult to apply. Alternatively, Coleman’s group deposited a MoS_2_ network film^6^ and detected a significant photoresponse without applying a gate voltage.

Although different devices have been made, the mechanisms of the photoresponse of MoS_2_ are still not fully understood, especially the two-step current response, which includes a fast and a slow increase in the current. It is generally believed that the fast rise of the current is due the separation of electron-hole pairs. However, the slow rise of the current has been explained by various mechanisms, such as a persistent photocurrent (PPC)^7^, trap recombination (TR)^6,8^ and the photothermoelectric effect (PTE)^9^. In addition to the mechanism, some factors that impact the application of MoS_2_ films as a photodetector are unclear, such as the minority-carrier diffusion length, the ideal thickness, and the geometry of MoS_2_ films.

In this work, we produced large-area MoS_2_ films with various thicknesses using a protocol that combines acid exfoliation, vacuum filtration, flash-light processing and polishing. Gold electrodes separated by various distances were deposited on the films for the photoresponse measurements. The in-depth study of the photoresponse of the MoS_2_ films indicated that the photoresponse is contributed by the photovoltaic (PV) effect and photothermal (PT) effect. The minority-carrier diffusion length was found to be approximately 1.36 µm, where the highest sensitivity to light was measured. This study explained the mechanism of the photoresponse of flash-light-processed MoS_2_ films and indicated the optimum data range for manufacturing photodetectors using such films, which could promote the application of MoS_2_ in optoelectronics.

## Experimental

Exfoliation of MoS_2_. A 0.1 g MoS_2_ (Sigma-Aldrich) sample was added to 300 ml of pH 1.0 HNO_3_ water solution, followed by the addition of 0.6 g of sodium dodecyl sulfate (SDS). The mixture was bath sonicated for 5 hours. The dispersion was left in the lab overnight to allow the large particles to settle out, leaving exfoliated flakes in the liquid phase. The exfoliated flakes were characterized using an atomic force microscope (AFM, VEECO Nanoscope IIIa), a transmission electron microscope (TEM, JEOL 2000FX), and X-ray diffraction (XRD, BRUKER D2 Phaser).

Coating of MoS_2_ films. Different amounts (10, 20, 40, 80, and 160 ml of dispersion) of exfoliated MoS_2_ dispersions were filtered through a polyvinylidene fluoride (PVDF) membrane (Millipore), followed by rinsing with water three times to remove residual SDS and nitric acid.

Flash-light processing of MoS_2_ films. The filtered MoS_2_ films on PVDF membranes were processed using a Profoto D1 Air 1000 Ws Monolight flash head. The films were processed with 10 flashes at 400 Ws, 10 flashes at 550 Ws, and 5 flashes at 700 Ws. The processed films were further polished with a curved steel piece.

Deposition of gold electrodes on processed MoS_2_ films. A patterned template made on polyethylene terephthalate (PET) was placed above the processed MoS_2_ films before the gold deposition. The template was designed so that the parallel electrodes would have the same width but various separation distances so that one could study the influence of the distance on the photocurrent of sintered MoS_2_ films. Forty-nanometer-thick gold electrodes were deposited on the films using a thermal evaporator.

Measurement of photocurrent. The photocurrent of processed MoS_2_ films between gold electrodes was measured under simulated sunlight using a Source Measure Unit card (National Instruments PXI-4132) and controlled with a LabView program. The photocurrent was measured at different light intensities and bias voltages. The photocurrent at a single wavelength of light was measured by placing a monochromator between the processed MoS_2_ films and the light source. Light intensities were measured using a photodiode-based meter (Thorlabs, PM100D).

## Results and Discussion

**Figure 1:**
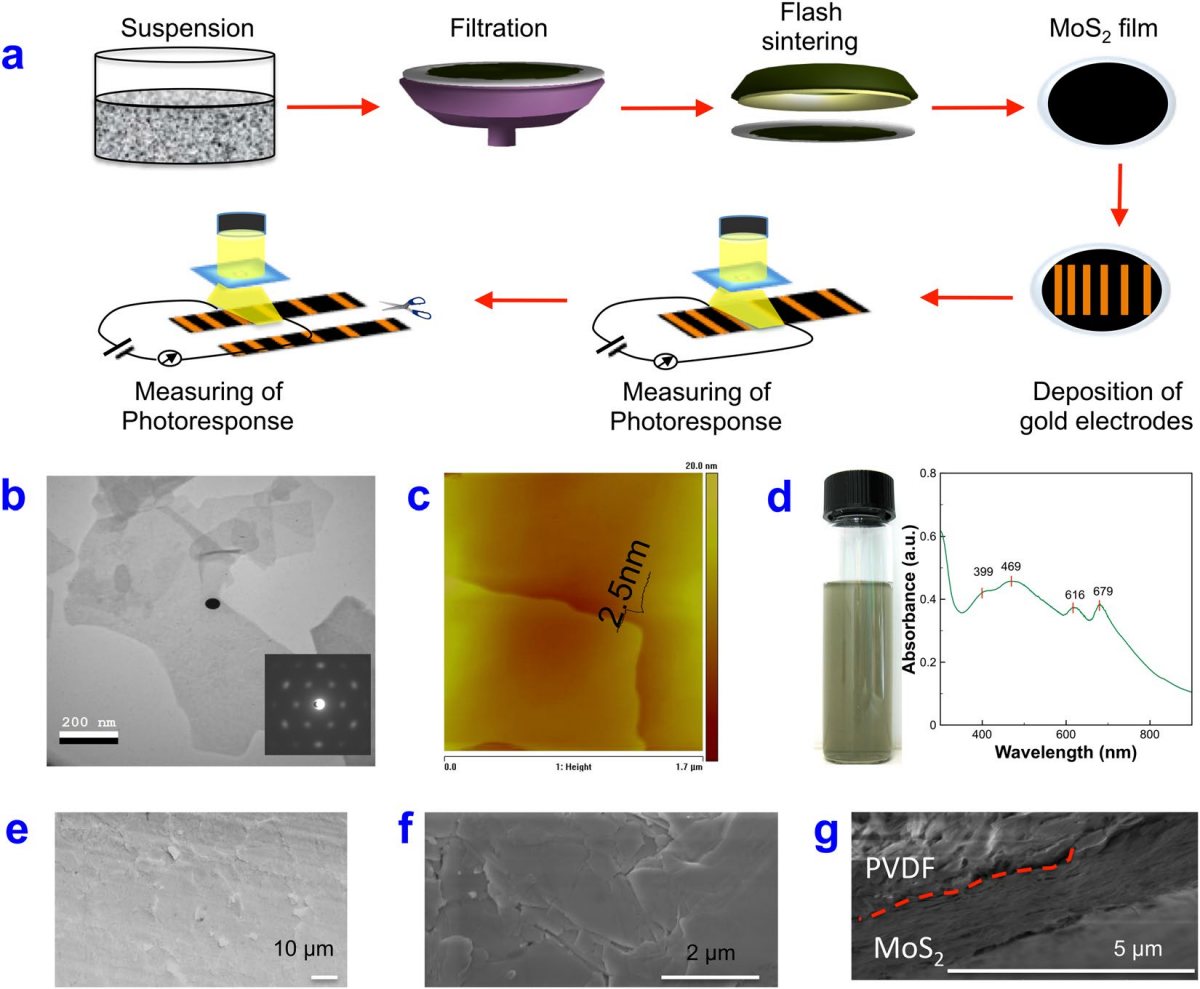
(**a**) Schematic drawing of experimental procedures. MoS_2_ was first exfoliated in a pH 1.0 nitric acid solution and then collected on a PVDF membrane by filtration, followed by flash-light processing and polishing. Gold electrodes with a designed pattern were then deposited onto the film, and the photoresponse was measured under illumination. (**b**) TEM image of exfoliated MoS2 sheets. The insert is the electron diffraction pattern of the flake. (**c**) AFM image of MoS_2_ sheets, (**d**) A photograph of exfoliated MoS_2_ after two months of settling and the UV/Vis absorbance of exfoliated MoS_2_ in water. Low (**e**) and high (**f**) magnification SEM images of MoS_2_ film deposited onto a PVDF membrane after flash-light processing and polishing. (**g**) Cross-section SEM image of MoS_2_ film.

The experiments were designed as shown in Fig. 1a. MoS_2_ was first exfoliated in a pH 1.0 nitric acid solution and then filtered and rinsed on PVDF films. The obtained films with various thicknesses were processed using flash-light, where the heated MoS_2_ bound well with the PVDF, which stabilized the films. The films were further polished with a metal plate, creating a smooth surface. The pressure added during the polishing process improved the contact between MoS_2_ flakes. After the films were prepared, patterned gold electrodes were deposited, where the distance between the electrodes was pre-designed. The photoresponse of MoS_2_ films between two adjacent electrodes was then measured. To further study the influence of the geometry of the MoS_2_ films, the films were cut into smaller pieces, and the photoresponse was measured again.

Exfoliation was performed in a pH 1.0 nitric acid solution^10^ to obtain thin MoS_2_ flakes. This method avoided the use of an organic solvent^11^ while allowing the production of a higher concentration of MoS_2_ flakes than is achievable in water^12^. The MoS_2_ sheets resulting from acid exfoliation were characterized by microscopy. Figure 1b shows a transmission electron microscope (TEM) image of acid-exfoliated MoS_2_. The average thickness of the flakes in the dispersion was approximately 2.5 nm, as measured using an AFM (Fig. 1c, supporting information Figures S1 and S2), while the thicknesses ranged from 1 to 8 nm.

Such thin MoS_2_ sheets were found to be stable in the exfoliating solution when large particles had settled out and had been removed after one week, leaving a yellow-green-colored dispersion (Fig. 1d). The dispersion was then transferred into another clean beaker and stored under the same condition for another two months, and no obvious sediment was observed, indicating a stable dispersion. Such a stable dispersion is due to the low pH value of the solution, where the pH of 1.0 is lower than the point-of-zero charge^13^ of MoS_2_, which is pH 2.0. Such a low pH value produces protons on the MoS_2_ surface that can prevent stacking of the MoS2 sheets.

We further characterized the dispersion with UV/Vis absorption (Fig. 1d), showing four characteristic absorption bands at 399, 469, 616, and 679 nm. The peaks at 616 and 679 nm represent the direct transition from the valance band to the conduction band at the *K-*point of the Brillouin zone, which are also called the B and A transitions^14,15^. Eda *et al*.^14^ and Posudievsky *et al*.^16^ noted that the good resolution of the A and B peaks suggests a significant structural order, indicating a successful exfoliation, which in turn is consistent with the microscopy results. The concentration of the MoS_2_ was 0.3 mg/ml, which was calculated from the UV/Vis absorption peak at 679 nm according to O’Neill *et al*.^17^.

To fabricate stable films, the MoS_2_ sheets in the solution were first filtered on a PVDF membrane with various volumes to control the thickness. Such filtration creates networks between the sheets^10^; however, the contacts were weak so the film was not stable. To enhance the stability, one can press the film; however, the pressed MoS_2_ film could easily fall off of the PVDF membrane. The solution is to create strong binding between the PVDF and the contacting MoS_2_ sheets and to then press the film. Such strong binding can be induced using flash-light-processing, as we have done in this work. The flash-light heats the MoS_2_, and the contacted PVDF forms strong contacts. After the processing, we hand polished the MoS_2_ film with a curved metal. The polishing created a smooth MoS_2_ film (Fig. 1e,f), and the pressure added by hand resulted in formation of good contact among the MoS_2_ sheets. Such procedure results horizontally stacked MoS_2_ flakes (Fig. 1g). XRD of the MoS_2_ film is give in supporting information as Figure S3. This process produced flexible and mechanically stable MoS_2_ films. Such films had a higher photoresponse than the films fabricated on glass without flash-light processing (Figure S4 in the supporting information).

**Figure 2:**
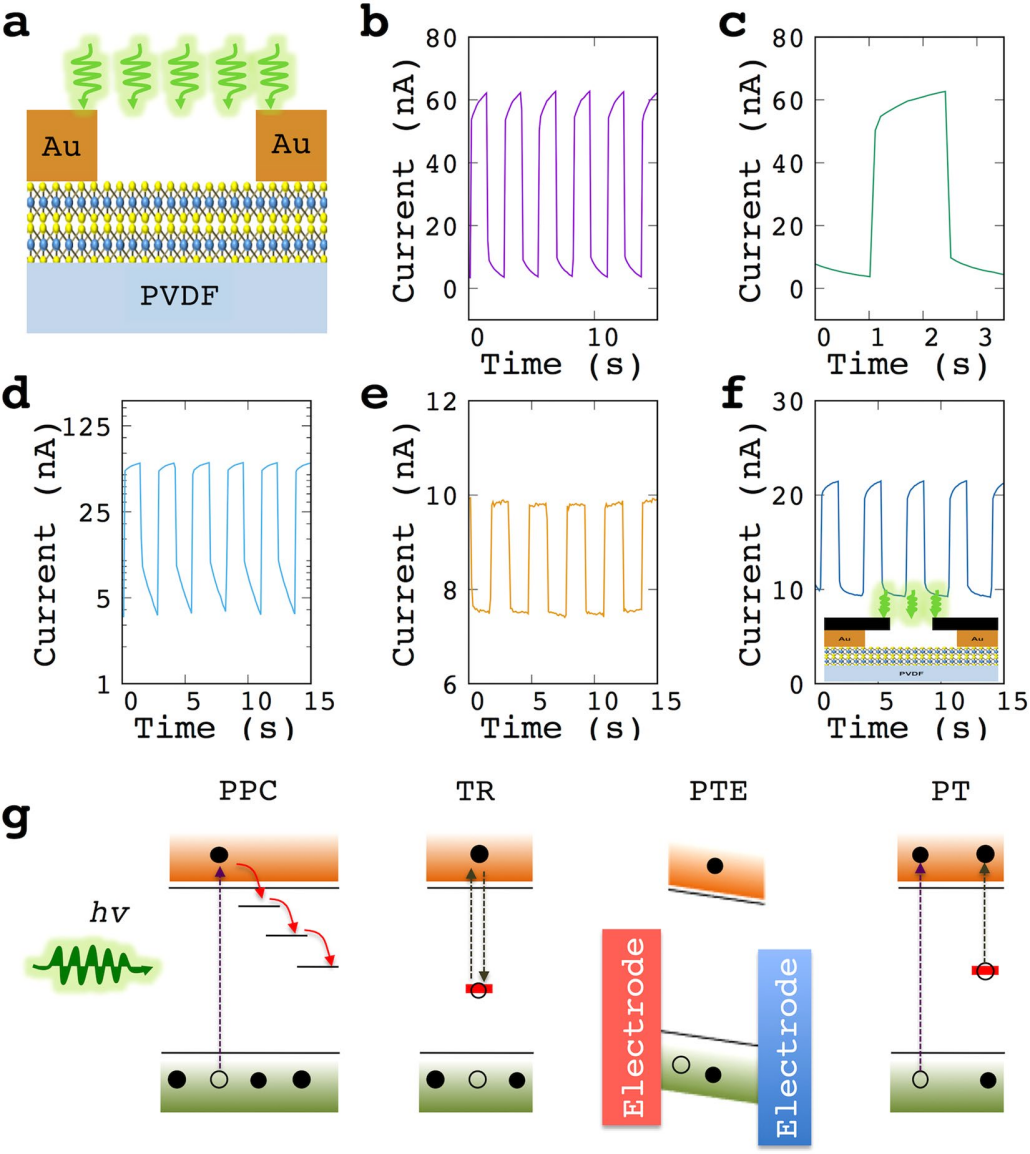
Photoresponse of MoS_2_ film. (**a**) A schematic drawing of the structure of the device, (**b**) Typical photoresponse of the MoS_2_ films, (**c**) Photoresponse of single cycle of pulse light, (**d**) Semi-log graph of (**c**,**e**) Photoresponse of MoS_2_ film on glass, (**f**) Photoresponse of MoS_2_ film when the electrodes were blocked from light, (**g**) Mechanisms that suggested for the slow current-rise of the photoresponse of MoS_2_ films, PPC: persistent photoconductance, TR: trap recombination, PTE: photothermoelectric effect, PT: photothermal effect.

**Figure 3:**
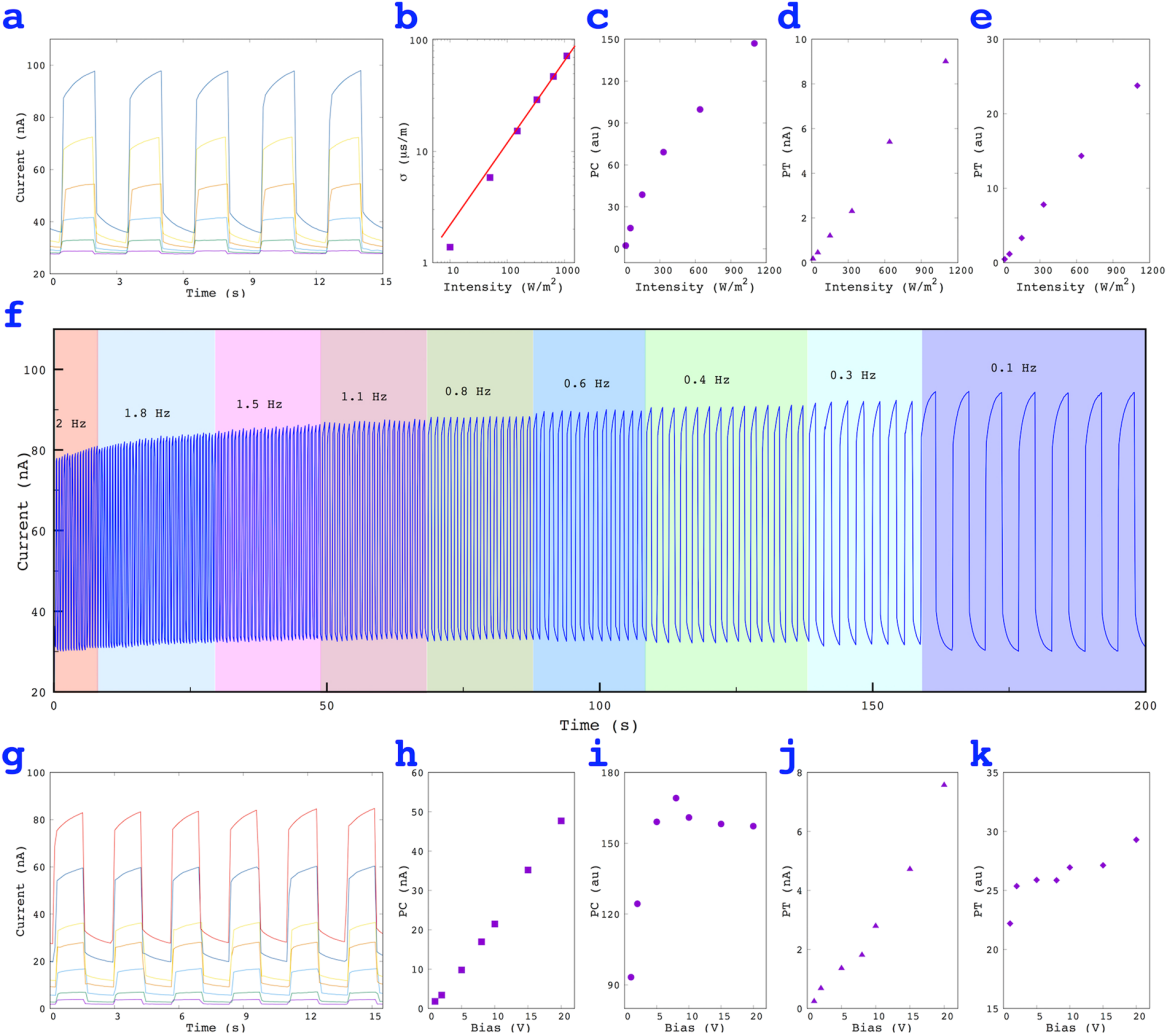
Photoresponse of a MoS_2_ film under various conditions. (**a**) Time-resolved photoresponse of a 10 × 2 mm film for various light intensities, (**b**) and (**c**) The absolute and normalized photocurrent change, PC (nA) and PC (au), (**d**) and (**e**) The absolute and normalized photothermal effect, PT (nA) and PT (au), (**f**) Time resolved photoresponse of a MoS_2_ film for various pulse light frequencies, (**g**) Time resolved photoresponse of a 10 × 2 mm film for various biases, (**h**) and (**i**) The absolute and normalized photocurrent change, PC (nA) and PC (au), (**j**) and (**k**) The absolute and normalized photothermal effect, PT (nA) and PT (au).

The photoresponse of a MoS_2_ film between pairs of gold electrodes (Fig. 2a) was measured with a Source Measure Unit (Fig. 2b). A typical current–time graph of a single light ON-OFF cycle is shown in Fig. 2c, indicating the fast and slow current rise steps. The fast rise in the current response is linked to electron-hole pair creation^18^, while the slow response was suggested elsewhere (Fig. 2g) to be due to persistent photoconductance (PPC)^7^, trap recombination (TR)^6,19^, or the photothermoelectric effect (PTE)^20^. However, these suggested mechanisms do not apply to our case. The semi-log graph (Fig. 2d) indicates that the current recovered very rapidly when the light was turned off, which is in contrast with the expected PPC effect behavior. It was also found that the length of the recovery time was the same as the irradiation time, regardless of how long the irradiation was (Fig. 3d), which is also unlikely for the PPC effect. Another suggested mechanism is TR^6,19^. However, our experimental results from the film on a glass substrate (Fig. 2e) suggested that TR could not be the reason in our case because the slow rise of the current was found to be sensitive to the substrates, whereas no such response of the current was found for the glass substrate (Fig. 2e). The third mechanism suggested by Perea-López and co-workers^20^ is the uneven photothermoelectric effect on both electrodes that contacted the MoS_2_ film. In our experiments, we tested the photoresponse of a MoS_2_ film while the electrodes were blocked from the light. The results (Fig. 2f) showed that the slow rise of the current remained, indicating that photothermoelectric effect is not the relevant mechanism.

Instead of the above mechanisms, we suggest that the slow process is due to the photothermal (PT) effect (Fig. 2g), which heats the MoS_2_ film, and subsequently, the heat excites electrons from the valence band to the conduction band. This is supported by experiment, since when a sample was heated by other means than light, a similar increase in the current was still observed (Figure S5 in the Supporting information). An effect caused by more electrons being excited from the valence band into the conduction band is a decrease in the resistivity. This decrease is reported as 0.2% for a 1 °C rise in temperature^21^. The film-heating explanation is further supported by the energy levels of the induced light being used. These energy levels are higher than the material’s band-gap energy, resulting in the excess energy being redistributed by a rapid relaxation process, which in turn heats the MoS_2_ film.

To further understand the PC and PT effects, we investigated the photoresponse on MoS_2_ films under different conditions. Figure 3a shows the current changes on a MoS_2_ film (0.72 µm thick) exposed to light with intensities of 10, 50, 150, 330, 640 and 1100 W/m^2^. The result shows clearly that both the PC and PT effects increased as the light intensity increased. Figure 3b illustrates the absolute current PC (*I*_*pc*_) and the light intensity (*P*) revealing a non-linear dependence and depicts a relationship of *I*_*pc*_ *~ P*^0.*77*^, indicating a trap-limited process^6,22^.

To show the sensitivity of a MoS_2_ film to light illumination, we normalized the change of the PC current using

1 $${\rm{PC}}({\rm{au}})=\frac{{{\rm{I}}}_{{\rm{pc}}}}{{{\rm{I}}}_{{\rm{dark}}}}\times 100 \% $$

and the resulting plot (Fig. 3b) shows that the PC (au) could achieve 150%, indicating a sensitive photoresponse.

The photothermal current was found to be non-linearly related to the light intensity (Fig. 3c), suggesting that trap recombination is not a significant factor of the slow increase in the current. In another report on the photoresponse of a MoS_2_ network film^6^, it was indicated that the slow current increased due to trap recombination and was independent of the light intensity. There, the MoS_2_ was deposited on a glass, which conducted the heat away from the MoS_2_, thus lessening the observed PT effect, while trap recombination played the primary role. However, PVDF has lower heat conductivity than glass and thus in our experiment, the PVDF did not conduct heat as well as glass did, caused an increase in the temperature of the MoS_2_ film and thus a significant PT effect. Similar to the PC (au), we plotted the response of a MoS_2_ film based on the PT effect, and the PT (au) value (Fig. 3c) indicated that the current change could reach 25% under 3 seconds of illumination.

The photoresponse was further studied at different pulse light frequencies (Fig. 3f). The results indicated that the PC current does not change significantly for different frequencies, while the PT current was found to be higher at low frequencies. Such results are inconsistent with the above suggestions, where at low frequencies, the longer illumination time on the MoS_2_ film leads to a higher temperature and a lower resistivity, which results in a higher current.

We further studied the influence of the bias voltage on the photoresponse of MoS_2_ films. The time resolved photocurrent is shown in Fig. 3g, and the relationship between the PC and the bias is illustrated in Fig. 3h, indicating that the absolute current increase was proportional to the bias. However, the plot of the PC (au) and the bias showed different results, as a linear current-bias dependency only occurred below 8 V (Fig. 3i). The reason for this difference was the increase in the background current (*I*_0_), since a higher bias produces a higher background current (Fig. 3g). A similar situation occurred when plotting the PT effect and the bias (Fig. 3j,k). No significant relation was found between the PT (au) and the bias, indicating that the PT response was less dependent on the bias.

**Figure 4:**
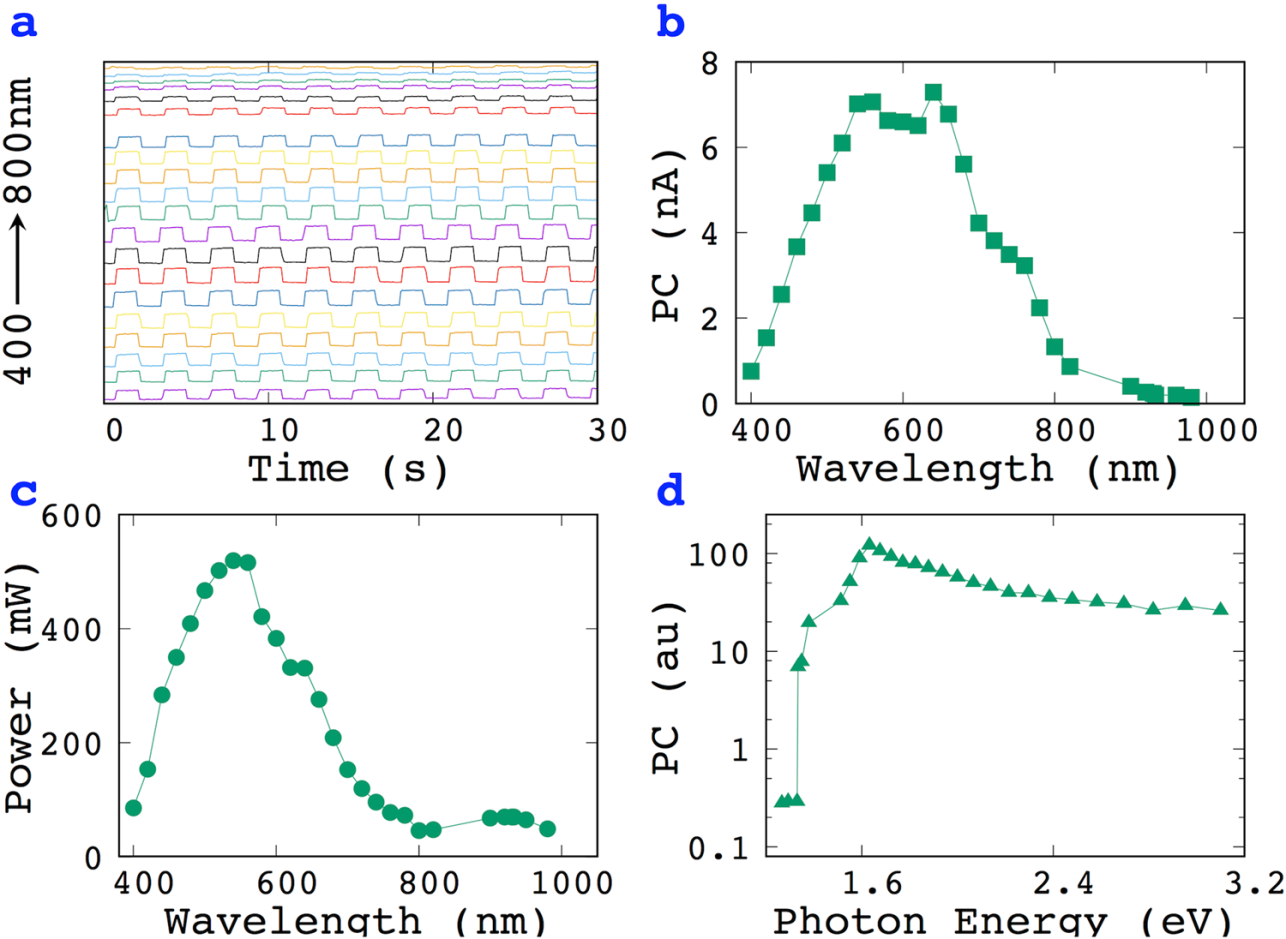
Photoresponse of a MoS_2_ film at various wavelengths where the light with different wavelengths was separated by a monochromator. (**a**) Time-resolved photoresponse of a MoS_2_ film, (**b**) Plot of PC versus wavelength, (**c**) The power of light from the monochromator, (**d**) Plotted PC (au) versus photon energy.

The photoresponse of the MoS_2_ film illuminated with various wavelengths was also studied. Figure 4a illustrates the time resolved photoresponse of a 10 × 2 mm MoS_2_ film, showing results from 400 to 800 nm with a 20 nm increment. The relationship between the PC and the wavelength is illustrated in Fig. 4b, which shows the results for 400 to 980 nm. Source-drain (IV) measurements on the film at each tested wavelength are given in Figure S6 in the supporting information. These results could be used to plot the photoresponse of a MoS_2_ film at different photon energies. However, one needs to normalize the PC at the various wavelengths with the detected light power (Fig. 4c) before plotting. Once this is done, the photoresponse of a MoS_2_ film for a photon energy can be illustrated, as in Fig. 4d, where a cut-off energy of 1.38 eV is observed, which corresponds to the indirect band gap of exfoliated MoS_2_^5^.

**Figure 5:**
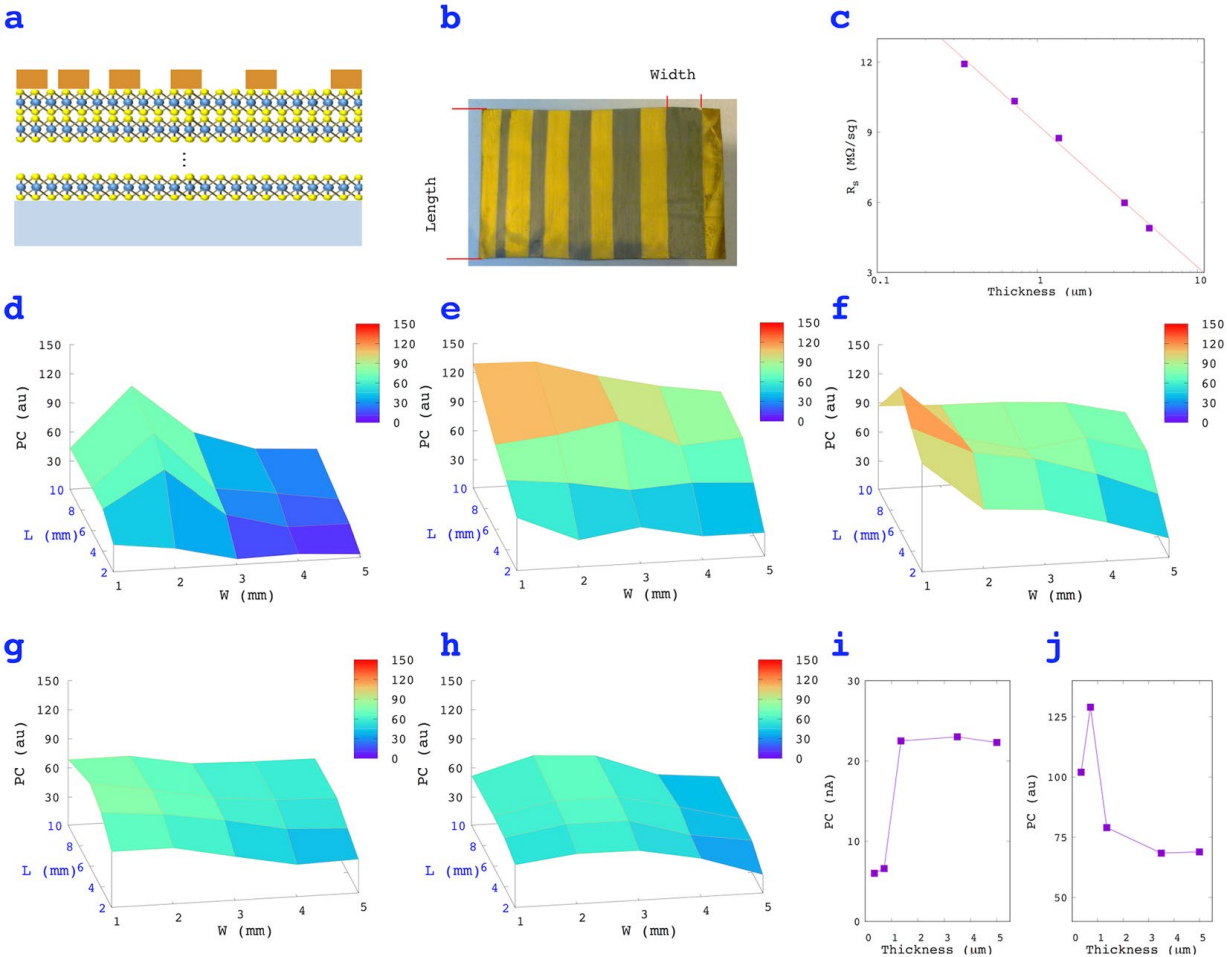
The influence of the geometry of a MoS_2_ film on the PC response. (**a**) A schematic drawing of the structure of the device, (**b**) a photograph of the device, (**c**) sheet resistance of films with various thicknesses, (**d**–**h**) maps of the PC (au) of MoS_2_ films with thicknesses of 0.35, 0.72, 1.36, 3.5, and 5 µm, (**i**,**j**) the absolute and normalized photocurrent change of 10 × 2 mm channels, PC (nA) and PC (au), at various thicknesses.

To further understand the photoresponse of MoS_2_ films, we prepared films with controlled thicknesses (*T*) and deposited gold electrodes with different distances between the electrodes (Fig. 5a). A photograph of a prepared device is shown in Fig. 5b. The width and length of the channels were later used to create maps of the photoresponse. Five films with different thicknesses (Fig. 5c) were selected for photoresponse measurements. The sheet resistances (*R*_*s*_) of the films were first measured before further measurements. The results showed an inversely proportional relationship between *R*_*s*_ and log (*T*).

Figure 5d–h show the PC (au) maps of MoS_2_ films with various thicknesses. The results showed the sensitivities of the photoresponse of large-area MoS_2_ films, which is important information for making photodetectors using MoS_2_ films. Fig. 5i is the plot of PC and PC (au) versus thickness for 10 × 2 mm channels. For thin films, the PC increased as the thickness increases. However, the current stayed at approximately 23 nA when the thickness exceeded 1.36 µm. The reason behind this is minority-carrier diffusion^23^. If the thickness is smaller than the minority-carrier diffusion length, all electron-hole pairs drift to the highly conductive surface channel^24^; thus, thicker films produce a higher current. If the thickness is greater than the minority-carrier diffusion length, the electron-hole pairs that are created over the free path will recombine before they reach the surface channel, thus only the pairs within one free path below the surface channel contribute to the PC. Our results suggested that the minority-carrier diffusion length was approximately 1.36 µm.

Despite the higher PC values of thick films, the sensitivities of thick films to light were found to actually be lower than those of thin films. The PC (au) value for a 0.72-µm-thick film could reach 130% (Fig. 5j), while films thicker than 1.36 µm had values lower than 80%. The sensitivity differences were due to the background current (*I*_*0*_), since the thicker films had lower resistances (Fig. 5c).

## Conclusions

In summary, the photoresponse of MoS_2_ films fabricated via flash light processing was studied, and the results indicated that the mechanism involved a combination of photovoltaic (PV) and photothermal (PT) effects. The PT effect heats the MoS_2_ films, leading to a lower resistivity and thus a higher current. This was observed as the slow current increased after the sharp one that was produced by the PV effect. The PV and PT effects under different light intensities and biases were further studied, showing different dependencies. The results of the photoresponse of MoS_2_ films with various thicknesses indicated that the minority-carrier diffusion length in MoS_2_ films is approximately 1.36 µm, and the highest sensitivity to light illumination was found to be at a thickness of 0.72 µm. The results in this work illustrate the mechanisms of the photoresponse of MoS_2_ multilayer films, which could promote the application of such films in optoelectronics.

## Electronic supplementary material

Supplementary information
