# Supplementary material for: Photoconductivity of acid exfoliated and flash-light-processed MoS2 films
Source: Sci Rep. 2018 Feb 19;8:3296. doi: 10.1038/s41598-018-21688-0 (PMC5818540; doi:10.1038/s41598-018-21688-0)
Supplement: Supplementary file 1 — Supplementary information [file 41598_2018_21688_MOESM1_ESM.doc]

Photoconductivity of acid exfoliated and flash-light-processed MoS_2_ films

Renyun Zhang, Magnus Hummelgård, Viviane Forsberg, Henrik Andersson, Magnus Engholm, Thomas Öhlund, Martin Olsen, Jonas Örtegren, and Håkan Olin


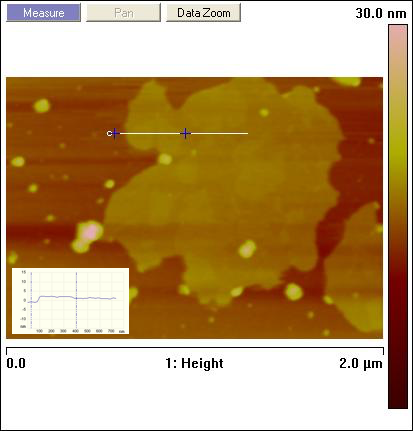


Figure S1. AFM of a MoS_2_ flake. The insert shows the thickness acroose the line.


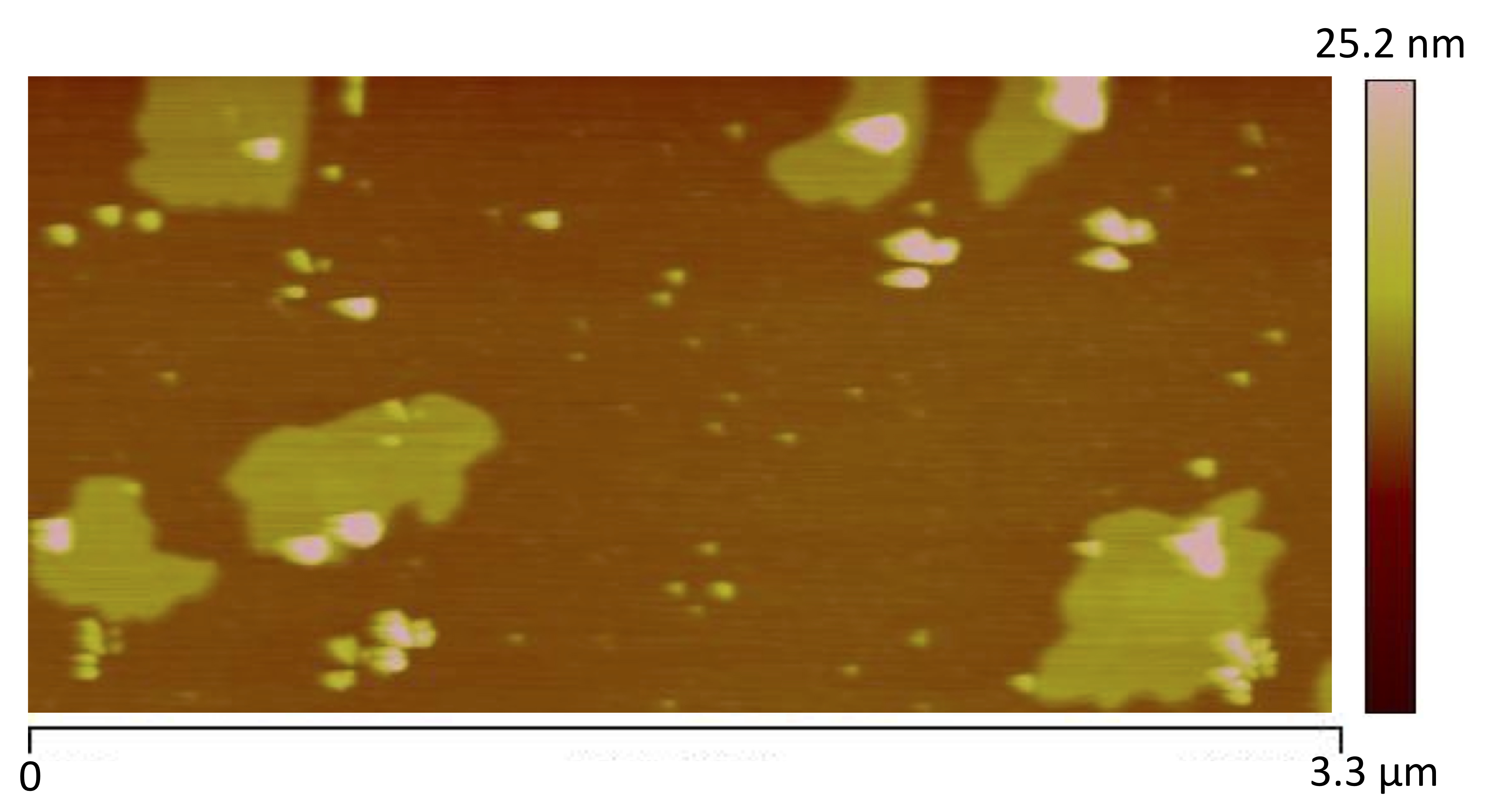


Figure S2. AFM of several MoS_2_ flake.


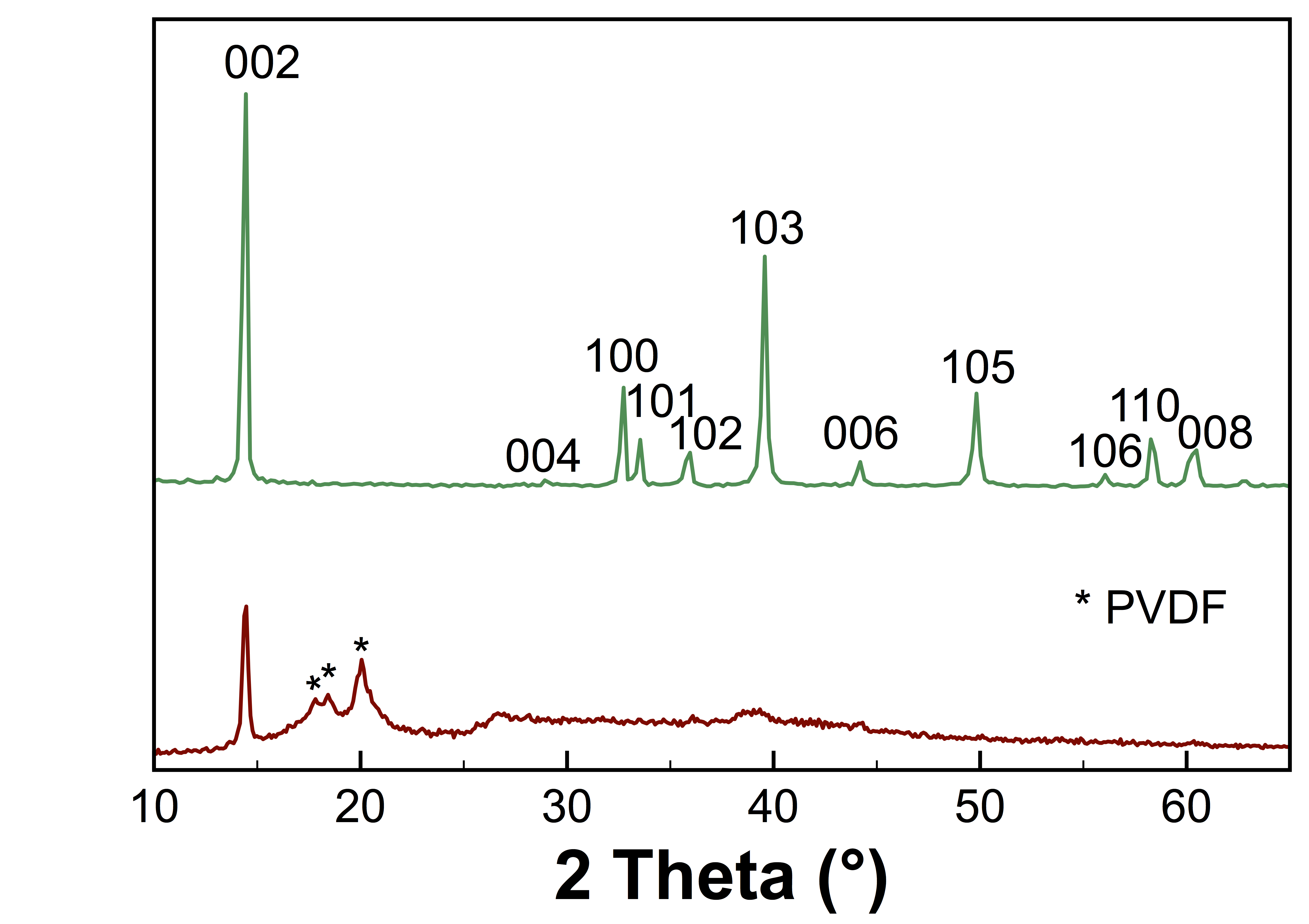


Figure S3. XRD of MoS_2_ film deposted on PVDF membrane. The top line shows the XRD of un-exfoliated MoS_2_ powder.


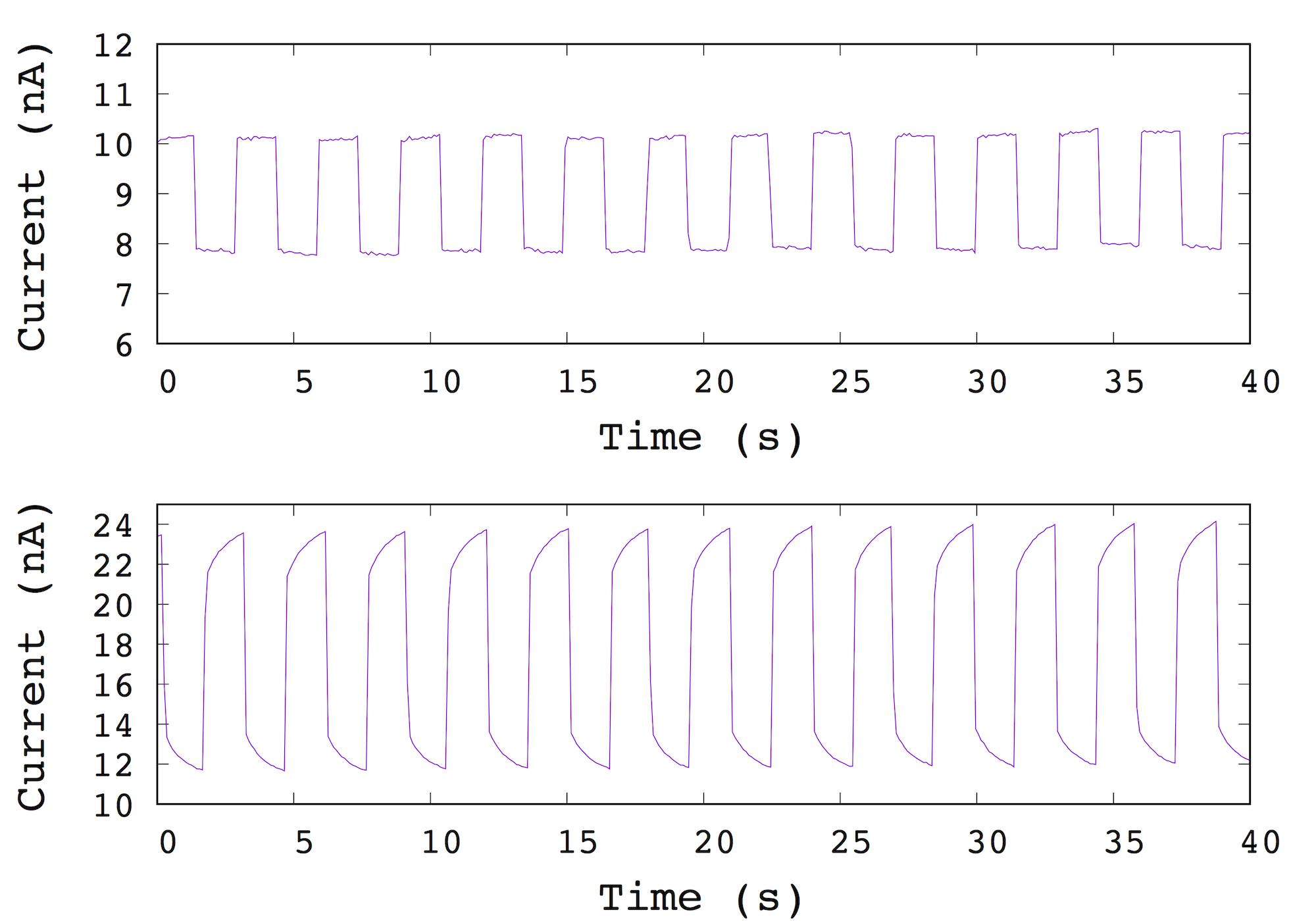


Figure S4. Photoresponse of MoS_2_ films deposited on glass (top) and PVDF (bottom).


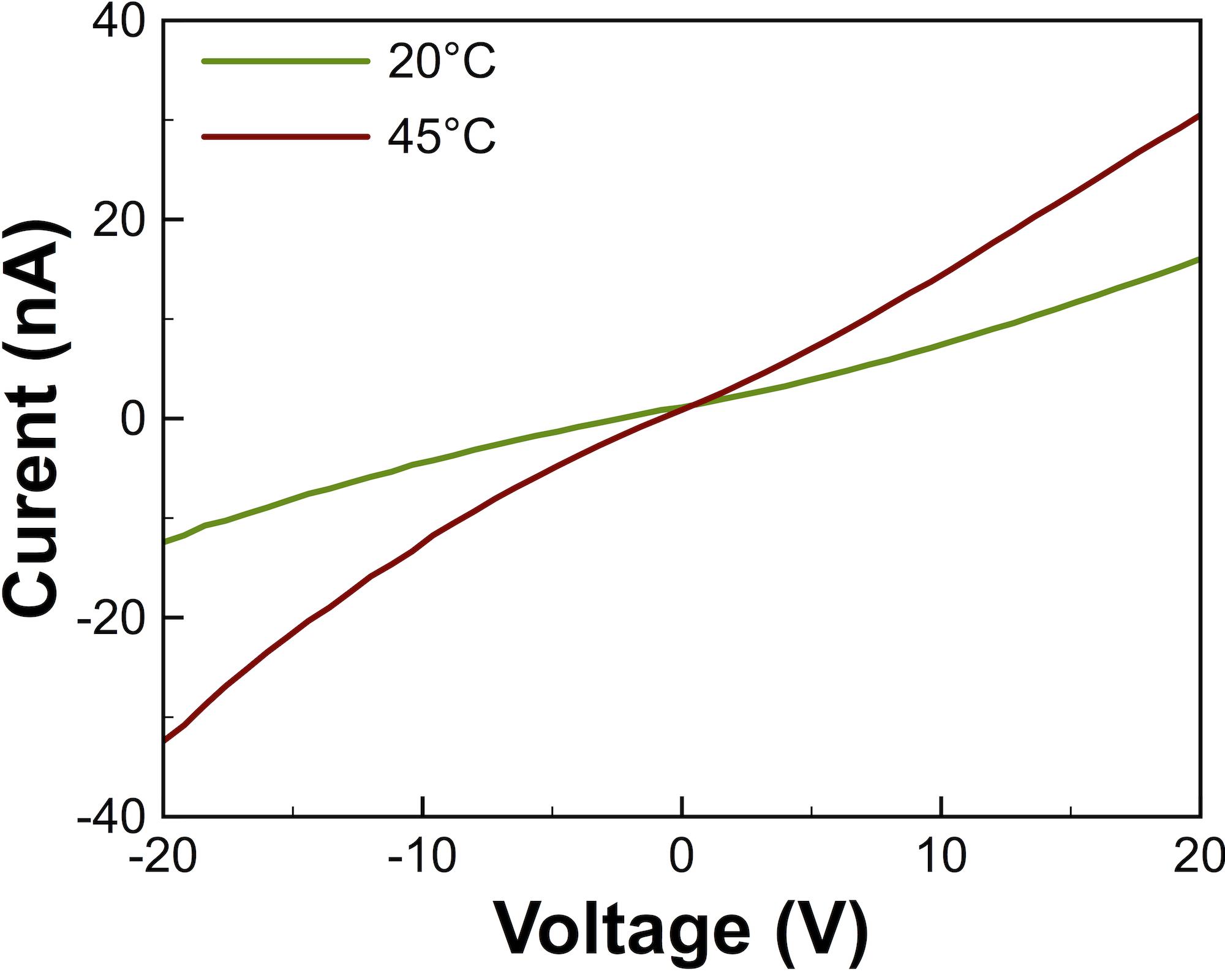


Figure S5. IV measurement of MoS_2_ film at different temperatures in the dark.


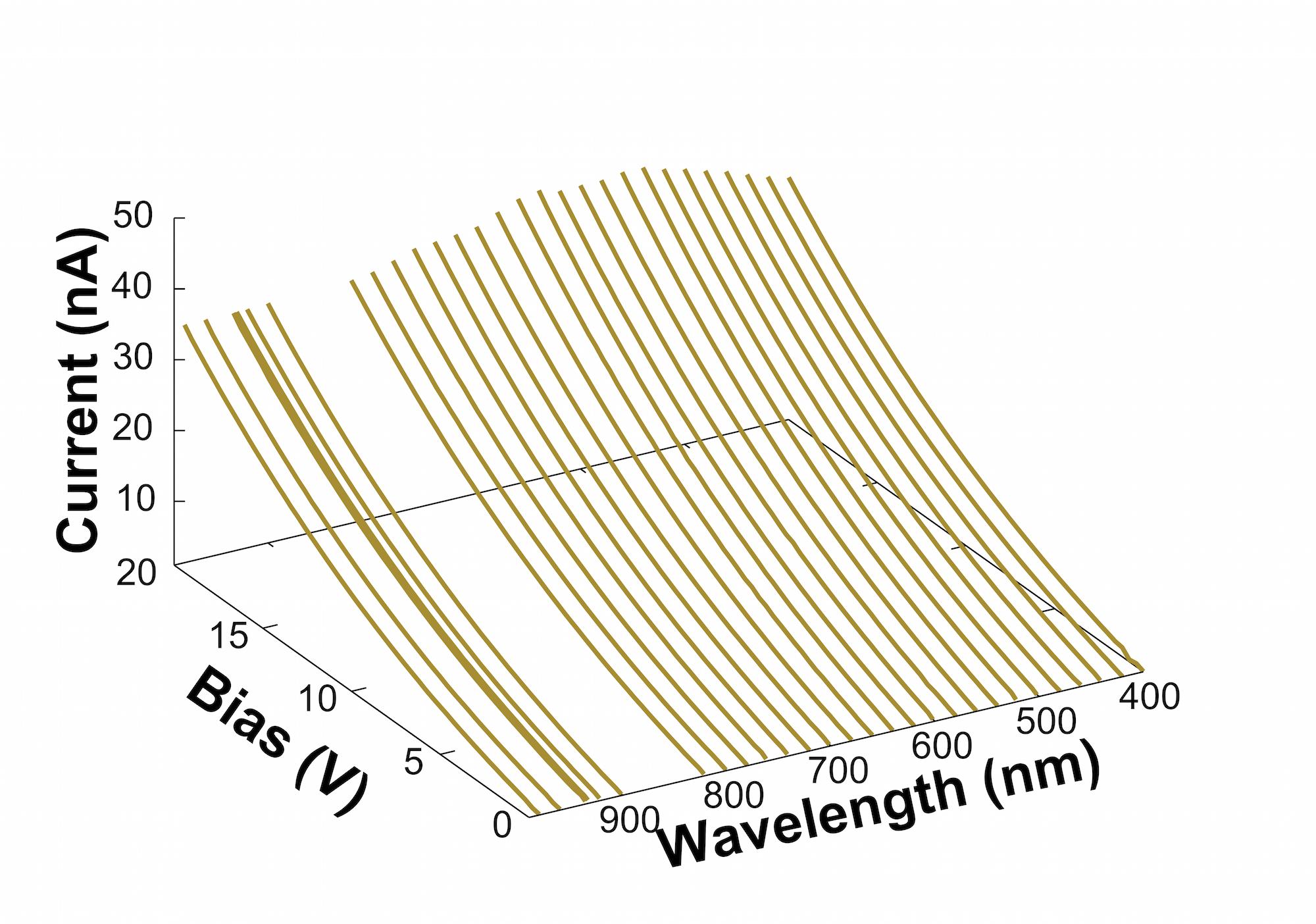


Figure S6. IV curves of a MoS_2_ film illuminated with light of various wavelengths.
